# *Strongyloides stercoralis* infection: A systematic review of endemic cases in Spain

**DOI:** 10.1371/journal.pntd.0007230

**Published:** 2019-03-12

**Authors:** Maria Barroso, Fernando Salvador, Adrián Sánchez-Montalvá, Pau Bosch-Nicolau, Israel Molina

**Affiliations:** 1 Primary Health Centre La Marina, Autonomous University of Barcelona, Barcelona, Spain; 2 Department of Infectious Diseases, Vall d'Hebron University Hospital, PROSICS Barcelona, Spain; Hitit University, Faculty of Medicine, TURKEY

## Abstract

**Background:**

*Strongyloides stercoralis* infection, a neglected tropical disease, is widely distributed. Autochthonous cases have been described in Spain, probably infected long time ago. In recent years the number of diagnosed cases has increased due to the growing number of immigrants, travelers and refugees, but endemically acquired cases in Spain remains undetermined.

**Methodology:**

We systematically searched the literature for references on endemic strongyloidiasis cases in Spain. The articles were required to describe *Strongyloides stercoralis* infection in at least one Spanish-born person without a history of travel to endemic areas and be published before 31st May 2018. Epidemiological data from patients was collected and described individually as well as risk factors to acquisition of the infection, diagnostic technique that lead to the diagnosis, presence of eosinophilia and clinical symptoms at diagnosis.

**Findings:**

Thirty-six studies were included, describing a total of 1083 patients with an average age of 68.3 years diagnosed with endemic strongyloidiasis in Spain. The vast majority of the cases were described in the province of Valencia (n = 1049). Two hundred and eight of the 251 (82.9%) patients in whom gender was reported were male, and most of them had current or past dedication to agriculture. Seventy percent had some kind of comorbidity. A decreasing trend in the diagnosed cases per year is observed from the end of last decade. However, there are still nefigw diagnoses of autochthonous cases of strongyloidiasis in Spain every year.

**Conclusions:**

With the data provided by this review it is likely that in Spain strongyloidiasis might have been underestimated. It is highly probable that the infection remains undiagnosed in many cases due to low clinical suspicion among Spanish population without recent travel history in which the contagion probably took place decades ago.

## Author summary

*S*. *stercoralis* is a soil transmitted helminth that is common in many subtropical and tropical countries, but is also found in other regions of the world. In this study we reviewed all published material on endemic infection of *S*. *stercoralis* acquired in Spain issued before 31st May 2018. We collected data from these articles and reported clinical and epidemiological characteristics of patients. Our systematic review of the articles showed a clear geographical pattern; nearly 97% of the cases described had been acquired in the Valencia province. Most of them (82.9%) were male, and most had current or past dedication to agriculture. Our results showed that 70.3% had at least one condition or treatment that could have made them more vulnerable to suffer a severe form of this helminthic disease. Our data suggests that *S*. *stercoralis* infection probably remains underdiagnosed in Spanish population. Due to the scarce information available about endemic strongyloidiasis in Spain until now, we believe that the present work will be relevant and the conclusions derived from it might raise awareness about underdiagnosis. Transmission risk factors described in the people diagnosed may be key for prevention and control strategies implementation.

## Introduction

Strongyloidiasis is a disease caused by soil-transmitted helminths, mainly by the species *Strongyloides stercoralis*. This intestinal nematode infects an estimated 300 million people worldwide, although this is probably underestimated. It is one of the most neglected of the neglected tropical diseases (NTD) and is widely distributed [1–2]. Although it generally occurs in subtropical and tropical countries, transmission is also possible in countries with temperate climates. Autochthonous cases have been described in Spain, possibly infected long time ago. It remains uncertain whether *S*. *stercoralis* is currently endemic in Spain. Still, some authors consider this country and some other southern European countries as endemic [3].

The life cycle of *S*. *stercoralis* is complex and follows multiple routes, including a complete life cycle outside the human host. The most frequent mechanism of infection is percutaneous entry of the filariform larvae. In healthy people, most of the cases are asymptomatic, although it can cause intermittent symptoms that mainly affect the intestine, the lungs or the skin [4].

About criterion used to establish the diagnosis of strongyloidiasis is not homogenized among the centers. The diagnostic laboratory criterion of strongyloidiasis is the observation of larval stages. However, in chronic infection, larvae excretion may be low and fluctuating, and microscopic observation is not sensitive enough and multiple stool specimens should be analyzed to increase the sensitivity of the method. The clinical criterion is a patient with epidemiological antecedents and any of the associated clinical manifestations, especially if it is an immunosuppressed patient.

These methods are laborious, time consuming, and in the case of fecal culture, requires well trained technicians in order to differentiate *S*. *stercoralis*. Several immunological tests have also been described (ELISA, IFAT and Western blot) with variable sensitivity and specificity depending on the population tested among other factors [1].

Alternative diagnostic methods, such as molecular biology techniques (mostly polymerase chain reaction, PCR) have been implemented. However, PCR might not be suitable for screening purpose, whereas it might have a role as a confirmatory test, since it still misses a relevant proportion of infected people [5].

Due to the subtle symptoms, low sensitivity of diagnostic techniques and the complex lifecycle that can cause asymptomatic autoinfection for decades, the prevalence of *S*. *stercoralis* is thought to be severely underestimated.

Typically risk factors for severe infection include immunosuppression, certain malignancies, human T-cell lymphotropic virus type 1 infection, and alcoholism. Likewise, *S*. *stercoralis* has been associated with agricultural or mining activities. In Germany, it was recognized as a parasitic professional disease in miners [6]. *S*. *stercoralis* infection has also been linked to low socioeconomic factors and infrastructure, indicating that it as a disease of disadvantage [7–8]. In recent years the number of diagnosed cases has been increasing in high income countries due to the growing number of immigrants, travelers and refugees [9–10].

To provide information on this topic, a systematic review of the cases of endemic strongyloidiasis in Spain was carried out, as well as the description of the epidemiological characteristics of these patients.

## Methods

Aiming to assemble all scientific articles based on endemic strongyloidiasis diagnosed in Spain, a systematic review was carried out. Relevant articles were retrieved from PubMed, EMBASE, Scielo, ISI Web of Knowledge, and Cochrane Library databases using combinations of the search terms adapted to each database. Additionally, Gray Literature in the form of communications presented at national congresses was performed, as well as OpenGrey. As a secondary source, Google Scholar and free internet search was used for non-indexed articles. The keywords were “*Strongyloides stercoralis”*, “soil-transmitted helminthiasis”, “endemic”, and “Spain”. The following combinations of MeSH were used in PubMed: (Strongy* [MeSH] AND Spain), ("Strongyloidiasis" [MeSH] AND Spain NOT "imported" NOT "immigrant"), and ("Strongyloidiasis" [MeSH] AND "endemic" AND "Spain").

The selection criteria were articles published in any language until May 31st 2018 that contained the description of at least one human case of infection with *S*. *stercoralis* acquired in Spain without a history of travel to endemic areas. No restrictions were applied based on the study design or data collection. Human filter was applied. A manual search of the bibliographical references cited in the relevant articles was carried out.

All potential articles were analyzed by two researchers to assess compliance with the selection criteria. In situations of missed information, the corresponding author of the paper was contacted to gather the information. If the author answered the required information to fulfill the inclusion criteria, those articles were considered. If not, they were excluded because they could not ensure the endemic acquisition.

The exclusion criteria included: animal studies, cases in which endemic infection could not be assured, cases of foreigners from an endemic country for *S*. *stercoralis*, native people with trips to endemic or probably endemic countries in the past (e.g. Italy, France or Portugal), transplanted people in which this contagion route could not be excluded, and duplicated cases.

Based on these criteria the articles were reviewed in two stages. In the first stage, articles were selected by titles and abstracts according to selection criteria. In the second stage, the full text of the articles was analyzed. Finally, the articles that met the selection criteria were included in the study.

From each study the following data was extracted: the study period, year of publication and number of endemic cases described. The following epidemiological data from patients described in the studies was collected: age, gender, geographical origin, medical comorbidities and concomitant treatments, occupation (or hobbies if relevant), other risk factors, year of diagnosis, diagnostic technique used for diagnosis, presence of eosinophilia and clinical symptoms.

## Results

**Fig 1:**
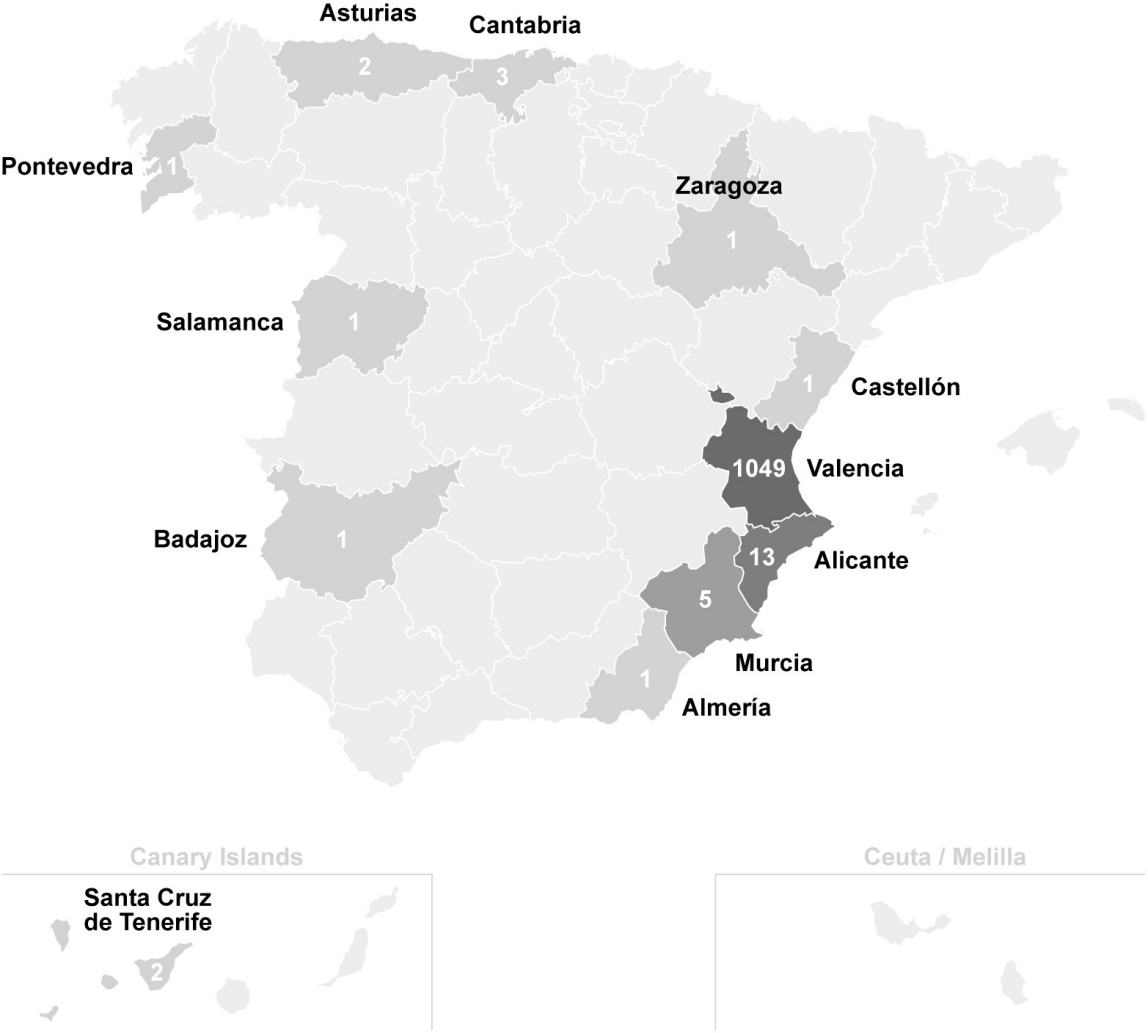
Geographical distribution of autochthonous *Strongyloides stercoralis* infection in Spain. The map was obtained from the open access website http://mapsvg.com/maps.

Thirty-six studies were included describing a total of 1083 patients with endemic strongyloidiasis in Spain (see Tables 1 and 2) [11–46]. The average age of the described cases was 68.35 years, ranging from 17 to 100 years old. Two hundred and eight of the 251 (82.9%) patients in whom gender was reported were male, and most of them had current or past dedication to agriculture. The province in whom most cases were described was Valencia, with 1049 people diagnosed. Alicante had 13 and Murcia 5, eventually describing cases in provinces of coastal oceanic climate with abundant rainfall most of the year and temperatures below 22°C (Cantabria, Asturias, and Pontevedra). See Fig 1.

**Table 1:** Main characteristics of included articles.

Reference	Study period	Number of cases	Year of publication
**Duvignaud et al [11]**		1	2016
**Valerio et al [12]**	01/2003–12/2012	2	2013
**Fernández Rodríguez et al [13]**		1	2012
**Mayayo et al [14]**		1	2005
**Oltra Alcaraz et al [15]**		261	2004
**Martinez-Vazquez et al [16]**		1	2003
**Román Sanchez et al [17]**		31	2003
**Román Sanchez et al [18]**		152	2001
Rodríguez Calabuig* et al [19]	10/1997–10/1999	15	2001
**Cremades Romero et al [20]**		1	1996
Rodríguez Calabuig* et al [21]	01/1994–06/1997	15	1998
**Llagunes et al [22]**		1	2010
**Esteve-Martínez et al [23]**		1	2013
**Olmos et al [24]**	1997	1	2004
**López Gaona et al [25]**		1	2009
**Escudero-Sanchez et al [26]**		1	2017
**Pacheco-Tenza et al [27]**	01/1999–03/2016	4	2016
**Esteban Ronda et al [28]**		1	2016
**Martinez-Perez et al [29]**	2000–2015	1	2018
**Igual Adell et al [30]**			2007
**Pretel Serrano et al [31]**	1994–1999	3	2001
**Ortiz Romero et al [32]**		1	2007
**Batista et al [33]**		1	1992
**Llenas García et al [34]**	01/1999–12/2017	9	2018
**Tornero et al [35]**	01/2002–12/2017	423	2018
**Corbacho Loarte et al [36]**	01/2008–12/2015	1	2017
**García García et al [37]**	2009–2010	2	2011
**Igual et al [38]**	2004–2005	112	2006
**Lozano Polo et al [39]**		1	2005
**Cremades Romero et al [40]**	04/1994–10/1995	32	1997
**Sanchis-Bayarri et al [41]**		1	1981
**Tirado et a [42]**		1	2002
**Nevado et al [43]**		1	1996
**Sampedro et al [44]**		1	1988
**Lopez Gallardo et al [45]**		1	1997
**Toldos et al [46]**		1	1995

* Data obtained through clarification of the main author

**Table 2:** Clinical and epidemiological characteristics of patients with autochtonous strongyloidiasis in Spain.

Gender and age (in years)	City and province of residence	Occupation	Comorbidity or concomitant treatment	Other risk factors	Year of diagnosis	Diagnostic techniques	Presence of eosinophilia. Eosinophils count per mm3. Percentage of eosinophils in white blood cell /percentage of patients with eosinophilia if relevant	Clinical symptoms	Ref.
W 17	La Safor, Valencia	No data	No data	Walking barefoot	2005	Fresh stool examination and/or fecal culture	Yes, 16,900	Abdominal pain, diarrhea and weight loss	[11]
W 61	No data	No data	Asthma and chronic eosinophilic pneumonia.	No data	2011	Serology	Yes 1,480 20%	Chronic urticaria and abdominal pain	[13]
M 79	Zaragoza, Aragón	No data	Mild malnutrition	No data	2004	Bronchial aspirate examination	No	Abdominal pain	[14]
261 M+W patients, 21–100 years old	La Safor, Valencia	124 agriculture activities, 18 construction activities	No data	33 irrigation ditches cleaners, 6 ditch baths	1995–1999	Fresh stool examination, Baermann test and fecal culture	No data	No data	[15]
M 25	Fornelos de Montes, Pontevedra	Logging company	No data	Occasional agricultural work and bathing in river	2002	Fresh stool examination, and examination after concentration (Ritchie)	Yes 22,000	Nonspecific skin lesions and abdominal pain	[16]
31 M patients, mean age 68.6 ± 8.0	Gandía, Valencia	Farmers	No data	Work barefoot	2003	Fresh stool examination and/or fecal culture	No data	No data	[17]
120 M and 32 W, mean age 67	Gandía, Valencia	Farmers and farmer´s wives	44 COPD(no further details), 38 heart disease, 7 solid neoplasia, and 1 HIV infection (no further details	99 work barefoot or were spouses of farmers, 13 drink non-potable water	1990–1997	Fresh stool examination and fecal culture	82% (n = 125) had eosinophilia median count: 1,357+-811 severe cases median count 694 +-309	41.65% (n = 63) were asymptomatic, 13% (n = 20) had hyperinfection syndrome or disseminated strongyloidiasis	[18]
15 patients, mean age 66 ± 10	Oliva, Valencia	68% farmers (rice and citric)	No data	No data	1997–1999	Fresh stool examination and fecal culture	100% (n = 15) patients had eosinophilia (>500 eosinophils)	49% cough, 47% pruritus, and 38% dyspepsia	[19]
M 70	Ribera Baixa, Valencia	Farmer	COPD (FEV1 48%) + corticosteroids	No data	1995	Fresh stool examination and/or fecal culture	Yes 12,600 34%	Nausea, vomits, weight loss and abdominal pain	[20]
15 M+W, mean age 65 (SD 11.5)	Oliva, Valencia	Farmers	No data	66.6% had some risk factor (work barefoot, drink non-potable water)	1994–1997	Fresh stool examination and/or fecal culture	No data	56.6% symptoms (12% cough)	[21]
M 76	Valencia Province	No data	Crohn´s disease + corticosteroids	No data	2009	Bronchoalveolar lavage	No	Fever, arthralgia, dyspnea	[22- 23]
M 57	Santander, Cantabria	No data	HIV infection CD4 42/mm3 Rheumatoid arthritis Corticosteroids+ Immunosuppression	No data	2003	Postmortem histopathological examination (trachea, lungs, ileum, cecum and pericolonic lymph nodes)	No	Anorexia, dysphagia, odynophagia, night sweats, weight loss, diarrhea, and acute respiratory distress	[24]
W 82	Restiello-Grado, Asturias	No data	No data	Gardening hobby	2008	Fresh stool examination and/or fecal culture	Yes 6,120 38%	Abdominal pain and diarrhea	[25]
M 85	Born in Extremadura, living in Madrid	Farmer	No data	No data	2017	Serology	No data	No data	[26]
M 69	Orihuela, Alicante	Farmer	Lung carcinoma, chemotherapy, inhaled corticosteroids	No data	2007	Sputum examination	Yes 600	Hemoptysis, hyperinfection syndrome	[27]
W 73	Redovan / Vega Baja del Segura. Alicante	Farmer	Diverticular disease	No data	2015	Serology	Yes 2,700	Abdominal pain and diarrhea	[27]
M 80	Orihuela, Alicante	Farmer	Bladder tumor	No data	2015	Fresh stool examination and/or fecal culture	Yes 700	Abdominal pain, pruritus	[27]
M 72	Orihuela, Alicante	Carrier	COPD (no further details)+ corticosteroids	Farmer's work in his free time	2015	Fecal culture, serology, histopathological examination (colon)biopsy)	Yes 2,400	Hyperinfection syndrome	[27]
M 84	Chella, Valencia*	Farmer	Gastrectomy, asplenia, malnutrition and treatment with corticosteroids	Walk barefoot	2016	Bronchial aspirate examination	No	Asthenia, dysphagia, low-grade fever, anorexia, and weight loss	[28]
M 40	Sta Cruz de Tenerife, Canarias	Construction worker	Immunosuppression	Walk barefoot in mud	2006	Fresh stool examination	Yes No further data	Hyperinfection syndrome	[29]
M 81	Vega del Segura, Murcia	Farmer	COPD (FEV1 42%)	Walk barefoot	1998	Fresh stool examination	Yes 1500 21%	Abdominal pain and pruritus	[31]
M 77	Vega del Segura, Murcia	Farmer	COPD(FEV1 30%)+ corticosteroids	Walk barefoot	1999	Fresh stool examination	Yes 1,100 11%	Bronchospasm, hemoptysis	[31]
M 82	Vega del Segura. Murcia	Farmer	COPD (no further details) + corticosteroids	Walk barefoot	1999	Fresh stool examination	Yes 3,900 29%	Dyspnea, wheezing, abdominal pain, meteorism, and pruritus	[31]
M 85	Murcia	No data	COPD (FEV1 50%) Corticosteroids for the last month	No data	2008	Bronchial aspirate examination	Yes 3,100 40.8%	Cough and dyspnea	[32]
M 35	Sta Cruz de Tenerife, Canarias	No data	HIV infection CD4 36	No data	1992	Fresh stool examination and/or fecal culture	No	Cough, fever, vomits, and diarrhea	[33]
8 M and 1 W, mean age 79	Orihuela, Alicante	6 farmers	2 neoplasia, 3 COPD (no further details) + corticosteroids	No data	1999–2017	Fresh stool examination and/or fecal culture, and serology	88.9% (n = 8) patients had eosinophilia median count 700		[34]
54 patients, mean age 72.6 (SD 9)	La Safor. Valencia	60% former rice farmers	No data	In 4 of them bathing in marshy waters for recreational reasons was assumed, or parents had worked on rice fields	2009:17 cases 2010: 12 cases 2011: 10 cases 2012: 5 cases 2013: 1 case 2014: 4 cases 2015:2 cases 2016: 1 case 2017: 2 cases	Fresh stool examination and/or fecal culture	No data	No data	[35]
No data	No data	No data	No data	No data	2008–2015	Harada Mori, Baerman and/or fecal culture	No data	No data	[36]
2 patients	Valencia. Valencia	No data	No data	No data	2009–2010	Fecal culture and Ritchie concentration method	No data	No data	[37]
112 patients	Gandía. Valencia	No data	No data	No data	2004–2005	Fresh stool examination and/or fecal culture	No data	No data	[38]
W 64	Santander, Cantabria	No data	Asthma + corticosteroids	No data	2005	Fresh stool examination and/or fecal culture	Yes 700 14%	Bilateral pleural effusion and respiratory failure	[39]
28 M + 4 W, mean age 68 (SD 7)	La Safor, Valencia	Farmers	62% of patients with comorbidities: COPD, asthma, alcoholism, diabetes mellitus, and neoplasia	Walk barefoot	1994–1995	Fresh stool examination and/or fecal culture, and bronchoalveolar lavage	100% (n = 32) had eosinophilia (>600)	65% had respiratory, digestive and/or cutaneous symptoms	[40]
M 71	Oliva. Valencia	Farmer	No data	Walk barefoot	1981	Cytological examination of an abdominal puncture, fresh stool examination and/or fecal culture	Yes 6,840 (38%)	Vomits and abdominal pain	[41]
M 71	Vinaroz, Castellón	Farmer	COPD (no further details) + corticosteroids	Walk barefoot	2002	Bronchial aspirate examination	No	Acute respiratory and renal failure, rash, and hemoptysis	[42]
W 63	Cantabria	No data	Asthma + corticosteroids	No data	1996	Fresh stool examination	Yes 19%	Dyspnea, cough, expectoration, diarrhea, vomits and pruritus	[43]
M 77	Oviedo, Asturias	Coal Miner	COPD (FEV1 30–50%) + inhaled corticosteroids and alcohol	No data	1988	Histopathological examination (duodenal biopsy)	No	Weight loss and gastrointestinal symptoms	[44]
M 71	Almería, Almería	No data	Ulcerative colitis + corticoids	No data	1997	Bronchial aspirate, stool and sputum examination, histopathological examination (colon biopsy)	No	Abdominal pain, diarrhea, fever, dyspnea, cough, and expectoration	[45]
M 58	Vega Media del Segura, Murcia	Farmer	COPD (no further details) + corticosteroids	No data	1994	Histopathological examination (duodenal biopsy), fecal culture	Yes 12,496 44%	Abdominal pain, diarrhea, vomits, and weight loss	[46]

* Data obtained through clarification of the main author.

**Fig 2:**
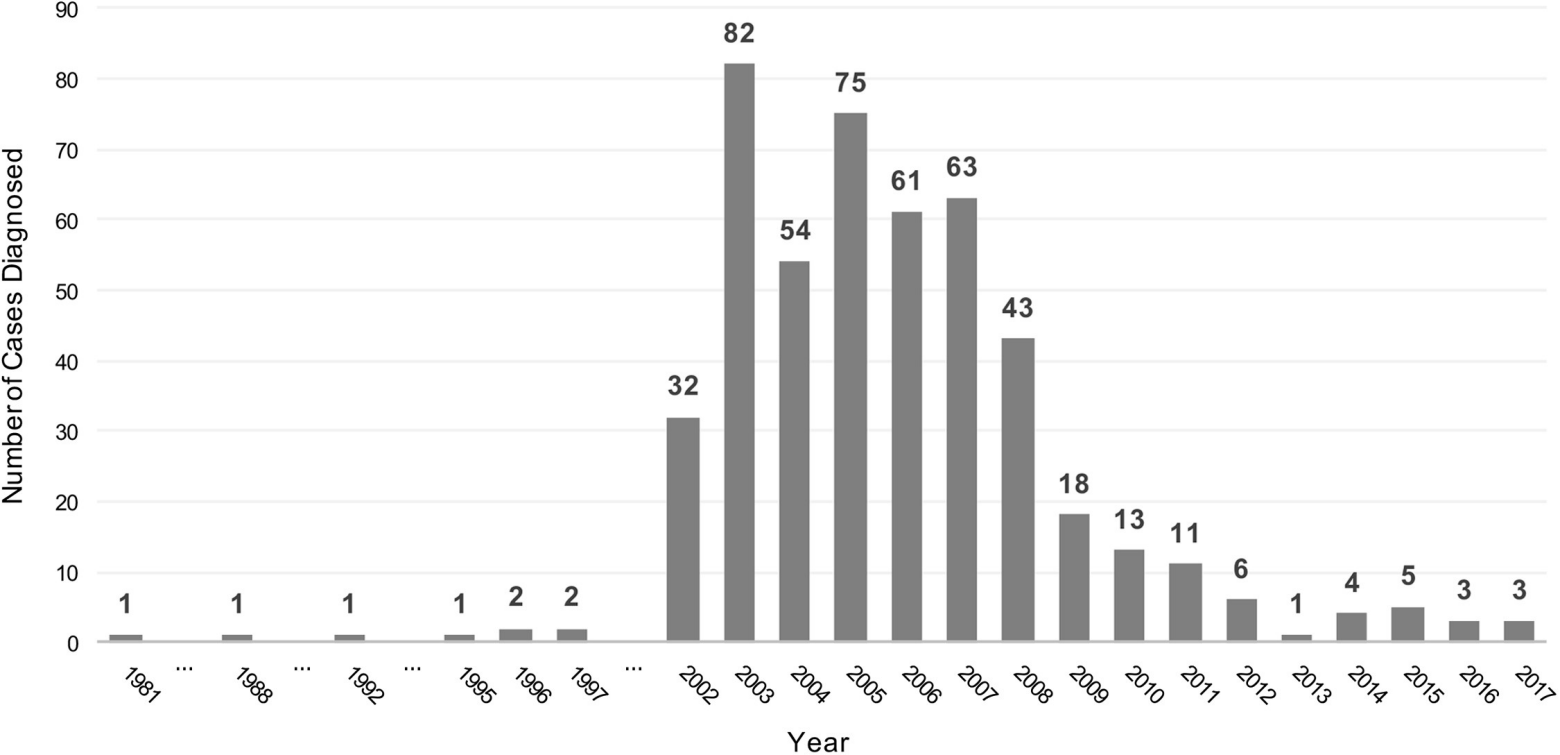
Number of patients diagnosed with autochthonous *Strongyloides stercoralis* infection in Spain per year. Only patients from articles that clearly specified the year of diagnosis of each case were included.

Regarding the number of diagnosed cases per year, a decreasing trend is observed since the beginning of this decade. The year with higher number of diagnosed cases was 2003, with 82 patients. Since 2011, no more than 10 cases have been reported annually (Fig 2).

The technique that led to the diagnosis of strongyloidiasis was described in 743 patients from twenty-six different articles. In some cases, different techniques were used for the same diagnosis. In 692 patients (93.1%), the technique used for the definitive diagnosis of strongyloidiasis was the fresh stool examination, specific fecal culture, the Baermann test, the Ritchie technique or the Harada Mori technique. In 39 patients (5.2%) the diagnosis was made by the sputum or bronchoalveolar lavage examination. In 6 cases (0.8%) the diagnosis was made by serological techniques and in another 6 cases (0.8%) the diagnosis was made by histopathological analysis.

In 26 of the 37 patients individually described, comorbidities were reported. Out of those, most frequent were diseases that associate the use of corticosteroids such as: chronic obstructive pulmonary disease (COPD), asthma, and inflammatory bowel diseases or immunosuppressive conditions due to advanced HIV infection (AIDS stage) or malignancies. In all patients diagnosed with COPD, severity of airflow limitation (FEV1) was according to the Global Initiative for Chronic Obstructive Lung Disease (GOLD) criteria at least moderate GOLD 2 (50% ≤ FEV1 < 80% predicted) if not severe: GOLD 3 (30% ≤ FEV1 < 50% predicted).

Overall, 70.3% of these patients had at least one comorbidity. In patients in whom blood test results were reported, 41 out of the 50 (82%) exhibited eosinophilia. The median eosinophil count in patients with eosinophilia was 4,057 eosinophil/mm3; considering 24 individual reported counts.

## Discussion

Strongyloidiasis prevalence may be underestimated in many countries. With the data provided by this review it is likely that underestimation could have been a reality for the last five decades in Spain. The main cause would be the lack of clinical suspicion. But also the subtle symptoms, the decades-long persistence of infection in untreated hosts and the absence of a diagnostic test of choice with high sensitivity and specificity would ultimately contribute.

An important finding of our work is that almost 97% of all published infections occurred in the province of Valencia. The fact that most cases diagnosed and published are in the province of Valencia, can respond to various reasons. Firstly, the area had the perfect combination of temperature and humidity, population exposed to *S*. *stercoralis* for occupational reasons such as rice farmers or irrigation ditch cleaners (activities that were characteristically carried out barefoot) and hygiene factors of rural areas during the 1960s (lack of drinking water and toilets in some homes). It is noteworthy that no cases of strongyloidiasis have been reported in other areas with similar climatology and population equally dedicated to the cultivation of rice fields, such as the Delta del Ebro in Tarragona province. We consider highly probable that there has been transmission in other areas outside those described. Secondly, health care professionals in the area of Valencia probably had a greater awareness of the infection, with a higher suspicion and therefore a higher number of diagnoses.

Although we concur that the estimated prevalence of *S*. *stercoralis* by one highly cited article is not representative of the entire country, we disagree that Spain should not be considered an endemic country [17]. However, autochthonous cases have been anecdotal in the last decade, as indicated by Martinez-Perez [47].

The results of the individuals diagnosed showed an average age close to 70 years old.

Given the known characteristics of the disease the contagion probably took place decades before the diagnosis, coinciding with the postwar period where hygienic conditions and infrastructure were affected. On one hand, factors of unavoidable mention that directly affect the transmission of this helminth are the improvement in hygienic conditions and the mechanization of agricultural work. On the other hand, the increase of awareness by health care workers, especially from the most affected communities, may have led to the diagnosis of new cases in recent years.

An overall higher incidence rate in male gender is described, which is consistent with previous studies [15, 17, 21, 27]. This might be explained due to a gender biased; since some articles focus on screening high risk population (farmers or smokers with COPD), traditionally associated with gender roles.

Regarding the diagnostic techniques used, there is great heterogeneity among the different studies. The sensitivity of techniques based on microscopy is not good enough, particularly in chronic infections. Serology is a useful tool but could overestimate the prevalence of the disease due to cross-reactivity with other nematode infections and its difficulty distinguishing recent and past (and cured) infections. However, current serological tests are specific enough and negativization or a decrease in the titers could be observed 6–12 month after treatment, making this tool very useful [48].

There are some limitations that have to be mentioned. Inevitably there are cases of strongyloidiasis that have not been written for publication. In addition, ten articles had to be excluded due to lack of information about travel history or did not comply with the minimum information required. Therefore, it is highly probable that there were more than 1083 cases. Lastly, given the characteristics of this review, it is possible that there are some duplicate cases in multiple description articles and described individually by another researcher.

In summary, there are still new diagnoses of autochthonous cases of strongyloidiasis in Spain every year, especially as occupational hazard in a specific Spanish region. Although the number of diagnoses is much lower than in the past decade, it is highly probable that the infection remains undiagnosed due to low clinical suspicion among Spanish population without recent travel history. Epidemiological studies in at risk areas based on serological techniques could give more information about the real situation of autochthonous cases of strongyloidiasis in Spain.

## Supporting information

S1 PRISMA Checklist. (DOC)

S2 PRISMA Flow diagram. (DOC)
