# Supplementary material for: Strongyloides stercoralis infection: A systematic review of endemic cases in Spain
Source: PLoS Negl Trop Dis. 2019 Mar 12;13(3):e0007230. doi: 10.1371/journal.pntd.0007230 (PMC6413904; doi:10.1371/journal.pntd.0007230)
Supplement: S2 PRISMA — (DOC) [file pntd.0007230.s002.doc]

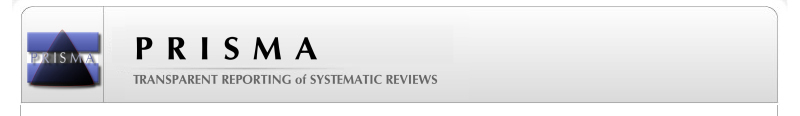
**PRISMA 2009 Flow Diagram**

**Screening**

**Included**

**Eligibility**

**Identification**

Records identified through database searching
(n = 123)

Additional records identified through other sources
(n = 23)

Records after duplicates removed
(n = 104)

Records screened
(n = 104)

Records excluded
(n = 54)

Full-text articles assessed for eligibility
(n = 50)

Full-text articles excluded, with reasons
(n = 14)

Studies included in qualitative synthesis
(n = 36)

Studies included in quantitative synthesis (meta-analysis)
(n = )
